# Supplementary material for: Methodological considerations in the assessment of direct and indirect costs of back pain: A systematic scoping review
Source: PLoS One. 2021 May 11;16(5):e0251406. doi: 10.1371/journal.pone.0251406 (PMC8112645; doi:10.1371/journal.pone.0251406)
Supplement: S1 File — (DOCX) [file pone.0251406.s001.docx]

**S1 File.** **Example of search strategy constructed with breakdown of hits obtained.**

| **MEDLINE** (1946 to February 2021), **Total hits:** 1,613 |
| --- |
| 1. exp Back Pain/ (34,476)  2. ((lumb* or spin* or vertebra*) adj5 pain).ti,ab. (13,336)  3. (backache or lumbago).ti,ab. (3,261)  4. (back adj5 (disorder* or ach*)).ti,ab. (1,742)  5. (back adj5 pain).ti,ab. (35,387)  6. or/1-5 (58,838)  7. exp "Costs and Cost Analysis"/ (217,015)  8. economic*.ti. (35,418)  9. cost*.ti. (93,479)  10. (health care adj5 (us* or utili* or expen*)).ti,ab. (33,098)  11. (financ* adj5 (burden or impact)).ti,ab. (5,222)  12. (resource* adj5 (us* or utili*)).ti,ab. (41,041)  13. (los* adj5 (product* or earn*)).ti,ab. (10,899)  14. ((sick* or absen*) adj5 expen*).ti,ab. (267)  15. or/7-14 (333,380)  16. 6 and 15 (1,733)  17. limit 16 to (english language and humans) (1,613) |
