# Supplementary material for: Methodological considerations in the assessment of direct and indirect costs of back pain: A systematic scoping review
Source: PLoS One. 2021 May 11;16(5):e0251406. doi: 10.1371/journal.pone.0251406 (PMC8112645; doi:10.1371/journal.pone.0251406)
Supplement: S2 File — (DOCX) [file pone.0251406.s002.docx]

**S2 File. Comparison of the direct and indirect costs of back pain and the cost components included.**

|  |  | **Direct costs** | | | **Indirect costs** | | |
| --- | --- | --- | --- | --- | --- | --- | --- |
| **Ref.** | **Country** | **National (million $)** | **Per capita ($)** | **Cost components included** | **National (million $)** | **Per capita ($)** | **Cost components included** |
| [37] | Sweden | 42 | 4.7 | Outpatient, inpatient, pharmaceutical | 217 | 24.4 | Absebteeism, early retirement |
| [39] | Sweden | 61 | 6.9 | Outpatient, inpatient, pharmaceutical, other | 346 | 38.9 | Absenteeism |
| [41] | Sweden | 261 | 27.7 | Outpatient, inpatient, pharmaceutical | 527 | 56.0 | Absebteeism, early retirement |
| [59] | Belgium | 302 | 29.5 | Outpatient, inpatient, pharmaceutical, other | 1,603 | 156.7 | Absenteeism |
| [44] | Netherlands | 622 | 38.0 | Outpatient, inpatient, pharmaceutical | 4,014 | 245.1 | Absebteeism, early retirement |
| [55] | Spain | 3,380 | 72.6 | Outpatient, inpatient, pharmaceutical, other | 9,878 | 212.3 | Absebteeism, presenteeism |
| [52] | Japan | 26,699 | 208.9 | Outpatient, inpatient, other | 11,866 | 92.8 | Absebteeism, presenteeism, early retirement |
| [61] | Canada | 832 | 28.6 | Outpatient, inpatient, pharmaceutical, other | 9,209 | 316.4 | Absenteeism, other |
| [15] | UK | 3,363 | 57.5 | Outpatient, inpatient, pharmaceutical, other | 22,015 | 86.7 | Absenteeism, other |
|  |  | 3,363 | 57.5 | Outpatient, inpatient, pharmaceutical, other | *10,358 | 177.1 | Absenteeism, other |
| [40] | Sweden | 130 | 14.8 | Outpatient, inpatient, pharmaceutical | 3,799 | 432.7 | Absenteeism, early retirement |
| [62] | Australia | 1,058 | 54.5 | Outpatient, inpatient, pharmaceutical, other | 8,400 | 432.8 | Absenteeism, other |
|  |  | 1,058 | 54.5 | Outpatient, inpatient, pharmaceutical, other | *5,220 | 268.9 | Absenteeism, other |
| [45] | Netherlands | 586 | 38.9 | Outpatient, inpatient, pharmaceutical, other | 7,319 | 485.7 | Absenteeism, early retirement |
| [42] | Netherlands | 6,101 | 377.8 | Outpatient, inpatient, pharmaceutical, other | 3,206 | 198.5 | Absenteeism |
| [57] | Switzerland | 2,109 | 283.5 | Outpatient, inpatient, pharmaceutical, other | 3,326 | 447.0 | Absebteeism, presenteeism, early retirement |
|  |  | 2,109 | 283.5 | Outpatient, inpatient, pharmaceutical, other | *1,785 | 239.9 | Absebteeism, presenteeism, early retirement |
| [50] | Germany | 33,176 | 402.3 | Outpatient, inpatient, pharmaceutical, other | 38,438 | 466.1 | Absenteeism |
| ** Estimated with alternative friction cost (fc) approach for the study above* | | | | | |  |  |
| *For the purpose of comparison, only studies that provided both direct and indirect national cost estimates* | | | | | | | |
| *were included in this table. All costs presented are in 2015 USD* | | | | | |  |  |
